# Allergen Immunotherapy with Depigmented–Polymerised Cat Allergoid Is Safe and Well-Tolerated in Patients with Allergic Rhinitis/Rhinoconjunctivitis

**DOI:** 10.3390/jcm14238456

**Published:** 2025-11-28

**Authors:** Ralph Mösges, Anna Rybachuk, Edmund Curtius, Cengizhan Acikel, Anne Drevermann, Nina Werkhäuser, Hacer Sahin, Nadine Katzke, Silke Allekotte, Ivo Landmann, Daniela Neumeyr, Eva-Cornelia Ticinelli, Angelika Sager

**Affiliations:** 1 ClinCompetence Cologne GmbH, 50668 Cologne, Germany; 2 LETI Pharma GmbH, 85737 Ismaning, Germany

**Keywords:** NIS-PASS, allergen-specific immunotherapy, allergic rhinoconjunctivitis, cat allergy, depigmented–polymerised cat allergoid

## Abstract

**Background/Objectives**: For decades, only native allergen extracts with a high incidence of adverse drug reactions (ADRs) were available for allergen-specific immunotherapy (AIT)—generally administered subcutaneously—to treat allergy to feline epithelia. Modified allergen extracts are a promising alternative to reduce the number of ADRs. The purpose of this study was to collect data on the safety of a depigmented–polymerised cat allergoid under real-world conditions in clinical routine. **Methods**: This study was designed as a voluntary non-interventional post-authorisation safety study (NIS-PASS), specifically focusing on adverse events (AEs)—including ADRs—that occur upon injection and assessing the influence on quality of life (QoL). The observation period was the initial phase (up-dosing) of the AIT. **Results**: Of 101 included patients, 91 patients were treated with the depigmented–polymerised cat allergoid. Regardless of the age group, around 50% of patients reported ADRs, which were mainly delayed local reactions (LRs). Other ADRs occurred only sporadically without persistent patient impairment. The ADR incidence did not differ significantly between quick and conventional up-dosing regimens or between adult and adolescent patient groups. The QoL data revealed no significant changes in any domains of the SF-12 questionnaire during the 12-week observation period. **Conclusions**: Overall, subcutaneous allergen immunotherapy (SCIT) with the depigmented–polymerised cat allergoid is a well-tolerated and safe treatment option for patients with cat allergy.

## 1. Introduction

Pets are the second most common cause of indoor allergies, after dust mites [1], including in Germany [2]. Sensitisation to pets is a risk factor for the development of allergic rhinitis/rhinoconjunctivitis (AR/ARC) and asthma [3], with cat epithelial allergy being one of the most common allergic diseases [4]. About 90% of cat allergy sufferers show an immunoglobulin E (IgE)-mediated reaction to Fel d 1, a protein produced by cats [2,5]. Fel d 1 is mainly produced by sebaceous gland cells and is present on the surface of the epidermis and fur [6]. Fel d 1 is found in saliva and transferred to the cat’s hair during grooming. The distribution of Fel d 1 in the environment is facilitated by cat hair, which acts as a vector for the allergen’s dispersion in the form of airborne particles [6].

IgE-mediated allergic reactions can be treated in one of three ways: (1) allergen avoidance; (2) symptomatic treatment (antihistamines, steroids, and bronchodilators); and (3) allergen-specific immunotherapy (AIT) [7]. Currently available AIT treatments are based on the use of native allergen extracts, administered subcutaneously (SCIT) or sublingually (SLIT). Clinical studies have demonstrated the efficacy of treatment with feline allergen extracts in patients suffering from cat allergy [8], with clinical efficacy correlated with the proportion of Fel d 1 [9]. Despite the proven clinical success with native extracts, high incidences of adverse allergic reactions are reported [10]. A sub-lingual tablet containing a monomeric cat allergoid is also available in some European countries; however, clinical evidence of its effectiveness is minimal [11].

Modified allergen extracts are a promising alternative to native allergen extracts to reduce adverse reactions to AIT. According to Carnes et al. (2018), allergoids are increasingly being applied as advanced products for allergy treatment because of new, beneficial mechanisms [12].

The depigmented–polymerised cat allergoid is manufactured by first producing a highly purified and concentrated allergen extract from the native extract during depigmentation and then polymerising it with glutaraldehyde [13]. These chemical modifications reduce allergenicity while maintaining immunogenic effects, thereby increasing the safety of the AIT [14].

In 2019, Mösges et al. conducted a meta-analysis evaluating the efficacy of AIT treatments containing depigmented–polymerised allergoids in patients with pollen- or house-dust-mite-induced rhinoconjunctivitis with or without allergic asthma [15]. Six double-blind placebo-controlled (DBPC) trials concerning pollen and two clinical trials involving house dust mites were analysed. AIT was shown to be more efficient in patients with more severe ARC symptoms compared to those with less severe symptoms, but efficacy was still demonstrated in the latter. Moreover, therapy with depigmented–polymerised allergoids did not increase the risk of local reactions (LRs) such as wheal and flare (odds ratio (OR): 1.55; 95% confidence interval (CI): 0.86–2.79) or systemic reactions (SRs) such as dyspnoea or generalised eczema (OR: 1.94, 95% CI: 0.98–3.84) compared to placebo.

In 2018, Dhami and Agarwal published a review evaluating the efficacy and safety of cat AIT treatments based on published studies [16], including only randomised DBPC trials. Overall, only three of the cited studies investigating subcutaneous cat AIT treatments reported AEs.

Following this comprehensive review of the available evidence, it was determined that AIT may be beneficial to some patients, particularly those with moderate to severe disease. Dhami and Agarwal concluded that further evidence was required. Health economic evaluations of cat-AIT were also deemed necessary [16].

The aim of the current study was to collect real-world data regarding the safety of a depigmented–polymerised cat allergoid in patients with ARC.

## 2. Materials and Methods

### 2.1. Study Design

The current study was a non-interventional post-authorisation safety study (NIS-PASS) investigating the safety of cat epithelial SCIT in everyday practice. The decision for the therapy had to be made before the inclusion in this safety study. For the purpose of generalisability, the plan was to conduct the study in approximately 80 study centres in Germany.

Sample size was calculated in SAS for Windows V.9.1. It was assumed that the proportion of local and systemic responses would be 16%, with a margin of error of 4%. A total of 404 patients are required for a 95% confidence interval, with a 20% probability of attrition. The total sample was drawn in a 1:3 ratio.

Based on the calculated sample size, the plan was to include 400 patients (300 adults, 100 adolescents) to detect age-specific differences in safety or tolerability.

During the treatment period, participating patients documented the presence or absence of AEs on the days of injection and the following two days in an electronic patient diary. The investigators transferred the information on AEs into the eCRF (electronic Case Record Form) following discussion with the patient, if applicable. At the start and end of the study, the impact of AR/ARC symptoms on the quality of life (QoL) was documented via the validated SF-12 questionnaire [17].

**Figure 1:**
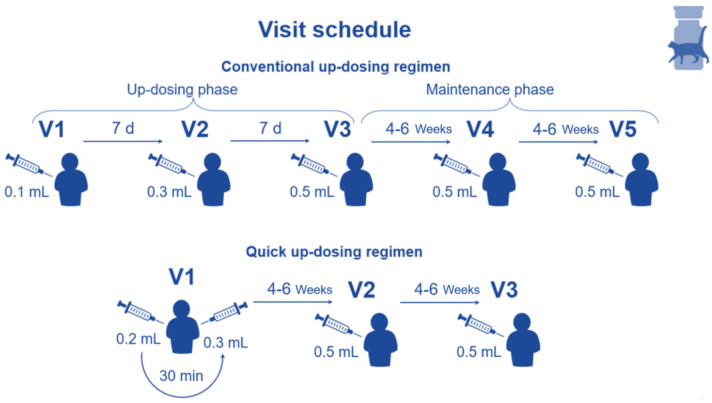
Visit schedule for the conventional and quick up-dosing regimens.

The depigmented–polymerised cat allergoid was administered via subcutaneous injection. Different dosing schemes (conventional or quick) were used according to the Summary of Product Characteristics (SmPC), as described later in this section and depicted in Figure 1. In this NIS-PASS, patients were observed during the initial 8–12 weeks of AIT, including the up-dosing phase. This time frame was chosen since the rates of ADRs are usually higher during the up-dosing compared to the maintenance phase.

### 2.2. Endpoints

The primary endpoints of the study were the number and severity of SRs categorised by the World Allergy Organization (WAO) criteria [18], the number and severity of LRs, and the onset of SR and/or LR (immediate or delayed).

The secondary endpoints of the study are listed below:

- Comparison of two up-dosing regimens (conventional up-dosing scheme (CUS) versus quick up-dosing scheme (QUS)) in terms of the primary variables;
- Comparison of CUS and QUS in terms of the proportion of patients reaching the maintenance treatment phase;
- Comparison of CUS and QUS in terms of the proportion of patients with LRs or SRs and the level of severity;
- Determination of changes in the QoL determined using the SF-12 questionnaire (assessment period: 1 week).

### 2.3. Setting and Subjects

Patients aged ≥12 years suffering from persistent moderate to severe cat-induced AR and/or ARC with or without controlled asthma (no exacerbations within the past 3 months), with an indication for AIT based on their symptoms and diagnostics, were eligible to participate in this NIS-PASS, in line with the SMPC of depigmented–polymerised cat allergoids.

Following standard clinical practice, the patient and physician agreed to initiate AIT with the depigmented–polymerised cat allergoid. Thereafter, patients were informed about the study and the associated data collection. Patients had to be able to understand the content of the study and—prior to enrolment—had to sign a declaration of consent form agreeing to the use of their data collected in the study. For adolescent patients, the declaration of consent form had to be signed by both the adolescent and the parent(s)/legal guardian(s).

The patient’s clinically relevant sensitisation to cats had to be demonstrated via a positive skin prick test (wheal diameter ≥ 3 mm) to *Felis domesticus* allergens.

Depending on the up-dosing regimen selected (conventional or quick), patients were observed for the initial 8–12 weeks of treatment with the depigmented–polymerised cat allergoid. Thereafter, the AIT for cat allergy was continued according to the SmPC and clinical routine practice.

AEs occurring immediately following injection were documented by the investigators, whereas delayed AEs experienced between 30 min and 48 h after injection were first documented by the patients in electronic patient diaries and subsequently evaluated and transferred to the eCRF by the investigators.

### 2.4. Study Flow

Patients could be treated in outpatient centres by investigators specialised in allergology. The up-dosing regimen used—either conventional or quick—was individually selected for each patient. In consequence, 3 (QUS, quick) or 5 study visits (CUS, conventional) were scheduled, with each patient participating in the study for 8–12 weeks (see Figure 1). In both cases, the up-dosing phase finished upon reaching the maximum injection dose of 0.5 mL, which is the start of the maintenance phase (see Figure 1).

Investigators were asked to adhere to the time schedules in accordance with the observational study plan. However, in accordance with the SmPC, time adjustments were acceptable if medically justified. After the 3rd (QUS) or 5th visit (CUS), the patient’s study participation ended, and AIT was continued following standard clinical practice.

### 2.5. Documentation of Adverse Events (AEs)

Study-specific reporting and documentation of AEs were performed via AE report form (paper-based), as well as electronically via the eCRF and an eDiary (electronic patient diary).

During the study, investigators were obliged to document

- All AEs occurring within 30 min after injection as ‘immediate’;
- All AEs occurring >30 min after injection as ‘delayed’.

On the days of injection and the 2 subsequent days, patients were asked to document any AEs in an electronic diary.

With AIT, most AEs observed during a study are indication-specific ADRs classified as one of the following:

1. Local reactions at the injection site (LRs);
2. Systemic reactions (SRs).

LRs were scored as immediate or delayed reactions, and the redness and swelling symptoms were further evaluated as mild, moderate, or severe according to the scoring scheme in Table 1. If only itching or pain at the injection site was observed as an LR (without concurrent wheal and/or redness), it was documented as a mild LR.

SRs were also scored as immediate or delayed reactions and their severity evaluated and ‘graded’ as Grade 1 to Grade 5 according to the WAO criteria (2010) [18].

### 2.6. SF-12 Questionnaire

During visit 1 and the last visit (visit 3 or visit 5, depending on the up-dosing scheme), patients aged ≥14 years were asked to complete a paper-based health-related QoL SF-12 questionnaire during an interview with the investigator.

The SF-12 questionnaire was developed and validated as an instrument to measure health-related QoL in adults and adolescents aged 14 years and older [19]. Based on 12 items, 8 aspects/dimensions are assessed: general health state/perception of health, physical capability, physical pain, physical ability to act, social capability, emotional ability to act, psychological well-being, and vitality. Adolescents aged 12–13 years did not complete a questionnaire.

### 2.7. Data Sources and Management

During the study, an electronic data capture system (EDC system) was used for data collection. Data was entered into the eCRF by the investigators or their study team.

The user concept of the secuTrial^®^ software, Version 6.4.0.13 (iAS, Berlin) ensured that only trained and authorised staff were permitted data access. The study-specific database stored in secuTrial^®^ is cloud-based and stored in Germany on the servers of Noris network AG, hosted by the company iAS. This company is ISO/IEC 20000- and ISO-27001-certified.

The CRO (Clinical Research Organisation) was responsible for processing the pseudonymised study data for scientific purposes. With the publication of results in the form of scientific presentations or publications, person-related patient data is guaranteed to be confidential.

While the CRO was processing the data, it was stored on servers hosted by the CRO and operated by the company Arwanet GmbH in Germany.

During the 1st patient visit for the study (Visit 1 (V1)), investigators were required to instruct participants on how to use the eDiary and provide them with the respective login data. Only in justifiable exceptions (e.g., absence of internet) were patients allowed to document their symptoms in paper diaries.

Data were collected in a pseudonymous manner. For this purpose, the participants received a patient identification number.

### 2.8. Statistical Methods

All statistical analyses were performed using IBM SPSS Statistics for Windows, version 27.0 or older (Armonk, NY, USA: IBM Corp.). The endpoints of the study were analysed with descriptive and exploratory statistics. Subgroups (in terms of gender, age, etc.) were analysed exploratively.

Continuous data were analysed using statistical ratios (mean, standard deviation, median, minimum, and maximum values). Categorical data were analysed using absolute frequencies and percentages of valid cases.

Confidence intervals were calculated using the Clopper–Pearson equation. Student’s *t*-test or the Mann–Whitney U test was used for continuous variables, and the Chi-square test or Fisher’s exact test was performed for group comparisons of categorical variables for exploratory purposes. The two-sided *p*-value for significance was set at 0.05.

No bias-reducing measures (such as blinding or a control group) could be implemented for this open-label observational study representing real world evidence.

### 2.9. Monitoring

To monitor the study conduct at the investigational sites, 3 on-site visits by the CRA per centre were planned for this NIS-PASS; 1 site initiation visit and up to 2 regular monitoring visits, depending on the site’s workload (e.g., no. of patients). During the site initiation visit, the CRA explained the study procedures and aims, provided training on the use of the eCRF, and explained how the study team should instruct the patients to use the eDiary. During the regular monitoring visits, the correctness and completeness of the declaration of consent forms, as well as the transfer of relevant data (especially AEs) from the patient file to the eCRF, were checked. In addition, the data entered in the eCRF were continuously monitored remotely, with a special focus on completeness and plausibility. The entries from the specific AE report forms (source data), copies of which had been sent to the CRO and the sponsor, were compared (100%) with the data in the eCRF.

### 2.10. Ethical Supervision

The study was entered in the EU PAS Register (EUPAS46091) and in the National study registry DRKS (DRKS00028182) in March 2022. All study documents were submitted to and approved by the responsible Ethics Committee of the University at Cologne (approval letter 21-1612_2-NIS dated 25 April 2022).

Where requested by the Ethics Committees of individual German federal states, specific patient information and declaration of consent forms were created.

## 3. Results

A total study duration of about 1.5 years was planned, but due to slow recruitment, the study period was slightly prolonged, ultimately lasting from May 2022 to April 2024. By the end of the recruitment phase in December 2023, only 101 patients out of 400 planned were enrolled in 22 outpatient allergy clinics in 7 of the 16 states of Germany. All these centres were required to have experience in conducting AIT studies.

**Figure 2:**
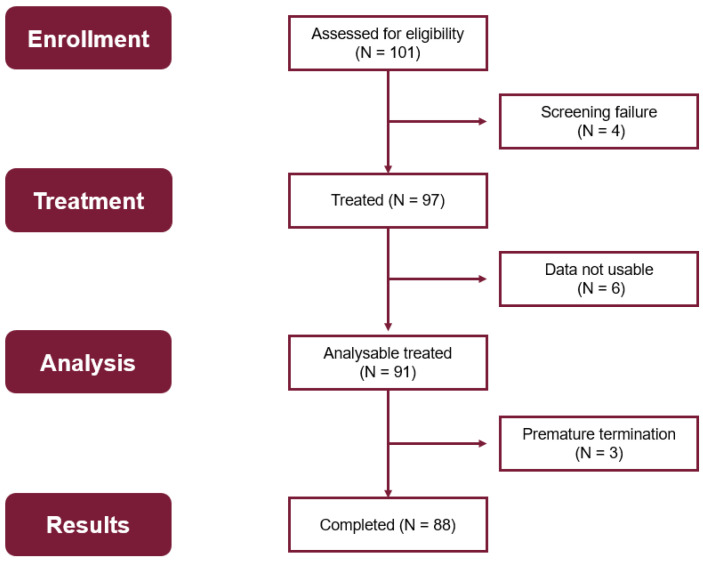
Overview of the patient populations of this study.

Figure 2 depicts an overview of the different patient populations for evaluation of the data. Of 101 patients enrolled, 97 patients were treated with at least one injection, and 4 patients failed screening. Data documentation was missing for 6 patients. Therefore, the Full Analysis Set comprises data from 91 patients (59 women and 32 men) who were treated with the study medication. The baseline demographic characteristics are shown in Table S2.

Three patients prematurely discontinued the study; they dropped out after V1 (one patient), during V2 (one patient), and after V4 (one patient) for tolerability reasons.

Eighty-eight patients completed the entire treatment course.

Overall, data for 9 adolescents (aged between 13 and 16 years) and 82 adults were analysed, of which 88 participants completed the study. The overall mean age of the adult study population was 34 years, ranging from 18 to 67 years.

Patients had the option of being treated with one of the two up-dosing treatment regimens according to the SmPC. Fifty-six (62%) patients selected the CUS, and thirty-five (38%) received the QUS. A total of 73.6% of the patients reported having at least one cat as a pet, with a maximum of four per household. These figures changed only marginally over the course of the study.

In addition to AR or ARC, 31.9% of the patients suffered from asthma. The majority of patients were polysensitised, with 63.9% also reacting to seasonal allergens, and 30% patients had been diagnosed with other perennial allergies.

During the study, 54% of the patients reported AEs; the AE frequency was very similar between adolescents and adults (56% and 54%), as shown in Table 2.

All but one participant reached the full maintenance dose, and 88 out of 91 treated participants completed the study as foreseen in the observational plan. The ratio of equal distribution (adolescents/adults) also corresponded to treatment-related AEs (=ADRs), with no significant differences in occurrence between adults and adolescents.

The incidence of ADRs did not differ significantly between the two up-dosing regimens, although more reactions were observed with the QUS.

The LRs were predominantly delayed, and the majority were mild (see Table 3).

In total, 41 related SRs—1 in an adolescent and 40 in adults—were reported in 25 patients during the study; SRs were predominantly Grade 2 for both immediate (seven SRs) and delayed (eight SRs) reactions (see Table 4). The majority of SRs were delayed but did not lead to adrenaline use or emergency medical intervention at home. One SR was categorised as Grade 2 by the investigator and documented as a serious adverse reaction (SAR) because dyspnoea occurred, but it resolved immediately. The narrative can be found in the Supplementary Material under Table S2. No Grade 3 or 4 SRs occurred.

The severity of ADRs was also compared between the CUS and QUS groups using adjusted age groups with ANOVA tests. Age-group-adjusted descriptive statistics and statistical analysis results were as follows: For LRs, immediate severity showed no significant difference between the CUS group (mean = 1.14, SD = 0.38, n = 7) and the QUS group (mean = 1.43, SD = 0.53, n = 7; *p* = 0.193). Similarly, delayed LR severity was comparable between groups (CUS: mean = 1.06, SD = 0.24, n = 17; QUS: mean = 1.00, SD = 0.00, n = 8; *p* = 1.000). For SRs, immediate grade data were limited with only two participants in the QUS group (mean = 2.00, SD = 0.00); statistical comparison was therefore not appropriate. Delayed SR grade showed a lower severity in the QUS group (mean = 1.50, SD = 0.58, n = 4) compared to the CUS group (mean = 2.00, SD = 0.00, n = 6), although this difference did not reach statistical significance (*p* = 0.089).

Most ADRs were local injection site reactions. Other ADRs occurred only occasionally and did not lead to any persistent patient impairments. They are listed in the Supplementary Material, Table S1.

The QoL data collected during the 8–12-week observation period did not reveal significant changes in any of the domains of the SF-12, as depicted in Table 5. One adolescent (13 years old) could not fill in the SF-12 due to being underage. The SF-12 was missing for one adult.

The comparison of the SF-12 values in the physical component score (PCS) and the mental component score (MCS) between baseline and the end of the observation period—i.e., after 8 or 12 weeks of AIT treatment—shows only marginal differences in both groups for the physical health dimension. On the other hand, adolescents who received the quick regimen experienced an approximately 20% apparent improvement in their mental health status (i.e., two times the standard deviation), as shown in Table 6.

## 4. Discussion

The German S2k guideline on allergen-specific immunotherapy [20] states that allergen avoidance should be the primary approach to managing cat allergy and that AIT should only be induced thereafter. This is, however, not a viable option for many cat owners (and lovers) or people who have frequent contact with cats. A safe and effective AIT for cat allergy would fill a gap for many of these desperate patients.

This NIS-PASS was designed within the regulatory framework of a voluntary PASS, following the European Network of Centres for Pharmacoepidemiology and Pharmacovigilance (ENCePP) Guide on Methodological Standards in Pharmacoepidemiology, thus meeting high methodological standards. The study was initiated immediately after the market launch of the product (depigmented–polymerised cat allergoid) in Germany and encountered a challenging medical–economic environment, where the reimbursement of the therapy for cat-owning patients was particularly questioned. It was therefore not surprising that the recruitment period had to be extended, and only around a quarter of the originally planned 400 patients were included.

The effort required of the study participants was minimal: completing the SF-12 questionnaire twice (approximately 2 min per questionnaire) and maintaining an eDiary on the days of injection and the two subsequent days (approximately 5 min per day). Their participation in the study helped improve the quality of safety data for the observed product. According to the German Medicinal Product Act AMG §4 (23) sentence 1, the NIS-PASS format was particularly suitable for collecting ‘real-world data’ in the context of routine clinical practice, which could not be detected in the defined and limited setting of a clinical trial.

Regardless of the age group, around 50% of patients reported ADRs, which were mainly delayed LRs (injection site reactions). Nevertheless, all but 1 of the 91 analysed participants reached the full maintenance dose, and 88 out of 91 treated patients completed the study in a regular fashion, with the other 3 participants dropping out for tolerability reasons. This is in clear contrast to other studies [21] reporting dropout rates of 20% or more. The distribution of LRs and SRs did not differ between the CUS and QUS groups, nor between adolescents and adults.

The number of reported SRs, all limited to Grades 1 and 2, was low compared to those reported in other studies [22]. These SRs primarily affected adults and were mostly delayed, thus supporting the good safety profile of the depigmented–polymerised cat allergoid.

In this study, among almost 100 patients, no emergency hospitalisation or use of adrenaline was reported, confirming a significantly better safety profile than with native allergens for subcutaneous application [22].

QoL did not improve significantly in the overall population during the relatively short observation period (up to three months). However, in the group of adolescents treated with the QUS, a clear improvement in mental health was observed. Given the small number of patients, this finding cannot be statistically confirmed. However, the observations made in this study correspond to those seen in a recently published real-world study in Spain [23] performed in 62 cat-allergic patients with the same AIT product. During 1 year of treatment, significant improvements in rhinitis and asthma symptoms were found. LRs were reported in 12.9% of patients, while systemic reactions were limited to Grade 1 and occurred in 11.3% of the patients. This underlines the beneficial safety and tolerability profile as well as the effectiveness of this form of cat-AIT, which had undergone extensive in vitro testing for efficacy and safety before it was introduced to the market [13].

Cat-SCIT is generally regarded as efficacious, as demonstrated by several studies.

In 2012, Patel et al. [24] published a randomised, double-blind, placebo-controlled parallel group study with Fel d1 peptide in patients with AR caused by cat allergy. They observed significant improvements in both nasal symptoms and eye complaints. The change was measured using the Total Rhinoconjunctivitis Symptom Score in patients treated with cat AIT. These patients had received four injections, either the peptide or a placebo, at four-week intervals. This improvement was still evident one year later [24]. In a double-blind, placebo-controlled study, Varney et al. [25] examined 28 patients with moderate to severe AR and asthma due to cat allergy and found a significant reduction in symptoms in the AIT group compared to the placebo group. They also observed a reduction in peak expiratory flow rate upon exposure to cats in the active group [25]. In a study of 28 monosensitised asthma patients aged 15 to 65 years, Alvarez-Cuesta et al. [26] investigated SCIT with polymerised cat allergoids compared to placebo. The study found significant differences in symptom and medication scores [26]. Cantani et al. reported significant differences in the total number of days with asthma symptoms and the need for asthma medication between SCIT for allergens and placebo in 20 monosensitised children [27]. The present study has some limitations. First, the results may not be generalisable, since fewer than 100 patients completed the study. This applies particularly to the adolescent age group, with just nine participants (=10%) in this subgroup of the total study population. Nevertheless, it is still one of the largest studies conducted with this allergen. A second limitation is the design of the study, which lacked a control group (e.g., placebo) and was unblinded. However, since the focus of this investigation was the safety and tolerability aspects of this recently marketed SIT for cat allergies, this type of study is generally accepted for the purpose. The selection of patients for one or the other up-dosing regimen was primarily based on the patients’ allergen reactivity—i.e., based on the available data from skin prick tests or specific IgE to the major allergen, Fel d1—but, of course, also based on considerations of practicability, patient preference, and medical risk tolerance. This resulted in clusters of treatment regimens in individual practices, which could naturally lead to a selection bias. The strength of this study is the well-defined approach of a PASS within the pharma-epidemiologic framework of the ENCePP.

To summarise, SCIT with a depigmented–polymerised cat allergoid containing a chemically modified allergen extract provides a well-tolerated and safe therapeutic option for patients with cat allergies. Long-term sustained effects of treatment lasting for at least 3 years and potential disease-modifying effects in the period thereafter need to be established.

## 5. Conclusions

This NIS-PASS, designed within the regulatory framework of a voluntary PASS, confirms the beneficial safety and tolerability profile during the initial phase of AIT treatment containing a depigmented–polymerised cat allergoid newly introduced to the German market. However, the short observation period (up to three months) limits conclusions on long-term efficacy and safety.

## Figures and Tables

**Table 1:** Evaluation of local reaction (LR) severity.

Severity of LR	Diameter of Wheal/Redness (of LR)
Mild	>0 to ≤5 cm
Moderate	>5 to ≤10 cm
Severe	>10 cm

**Table 2:** Presence of at least one adverse event (AE) (number of patients).

		Presence of at Least One AE		
		No n (%)	Yes n (%)	Total n (%)
**Adolescents**	CUS *	1 (20%)	4 (80%)	5 (56%)
	QUS *	3 (75%)	1 (25%)	4 (44%)
	Total	4 (44%)	5 (56%)	9 (100%)
**Adults**	CUS	22 (43%)	29 (57%)	51 (62%)
	QUS	16 (52%)	15 (48%)	31 (38%)
	Total	38 (46%)	44 (54%)	82 (100%)
**Total**	CUS	23 (41%)	33 (59%)	56 (62%)
	QUS	19 (54%)	16 (46%)	35 (38%)
	Total	42 (46%)	49 (54%)	91 (100%)
*p*-value	*p* = 0.914 **			

* CUS = Conventional up-dosing scheme/QUS = quick up-dosing scheme; ** Chi-Square test comparing incidence of AEs in adolescents with adults.

**Table 3:** Comparison of number and severity of local reactions (LRs) (number and % of patients).

	Severity of Immediate LR No. and % of Patients (n (%))				Severity of Delayed LR No. and % of Patients (n (%))			
	Mild	Moderate	Severe	Total	Mild	Moderate	Severe	Total
**Adolescents**								
CUS *	0 (0%)	0 (0%)	0 (0%)	0 0(0%)	2 (67%)	1 (33%)	0 (0%)	3 (75%)
QUS *	1 (100%)	0 (0%)	0 (0%)	1 (100%)	0 (0%)	0 (0%)	1 (100%)	1 (25%)
Total	1 (100%)	0 (0%)	0 (0%)	1 (100%)	2 (50%)	1 (25%)	1 (25%)	4 (100%)
**Adults**								
CUS	8 (67%)	4 (33%)	0 (0%)	12 (60%)	17 (71%)	5 (21%)	2 (8%)	24 (67%)
QUS	3 (38%)	4 (50%)	1 (13%)	8 (40%)	11 (92%)	1 (8%)	0 (0%)	12 (33%)
Total	11 (55%)	8 (40%)	1 (5%)	20 (100%)	28 (78%)	6 (17%)	2 (6%)	36 (100%)
**Total**								
CUS	8 (67%)	4 (33%)	0 (0%)	12 (57%)	19 (70%)	6 (22%)	2 (7%)	27 (68%)
QUS	4 (44%)	4 (44%)	1 (11%)	9 (43%)	11 (85%)	1 (8%)	1 (8%)	13 (33%)
**Total**	12 (57%)	8 (38%)	1 (5%)	21 (100%)	30 (75%)	7 (18%)	3 (8%)	40 (100%)
*p*-value	*p* = 0.394 **				*p* = 0.190 **			

* CUS = Conventional up-dosing scheme/QUS = quick up-dosing scheme; ** Chi-Square test comparing incidence of AEs in adolescents with adults.

**Table 4:** Comparison of number and grade of systemic reactions (SRs) (number of patients).

	Grade of Immediate SR No. and % of Patients (n (%))			Grade of Delayed SR No. and % of Patients (n (%))		
	Grade 1	Grade 2	Total	Grade 1	Grade 2	Total
**Adolescents**						
CUS	0 (0%)	0 (0%)	0 (0%)	0 (0%)	1 (100%)	1 (100%)
QUS	0 (0%)	0 (0%)	0 (0%)	0 (0%)	0 (0%)	0 (0%)
Total	0 (0%)	0 (0%)	0 (0%)	0 (0%)	1 (100%)	1 (100%)
**Adults**						
CUS	1 (25%)	3 (75%)	4 (50%)	7 (58%)	5 (42%)	12 (75%)
QUS	0 (0%)	4 (100%)	4 (50%)	2 (50%)	2 (50%)	4 (25%)
Total	1 (13%)	7 (88%)	8 (100%)	9 (56%)	7 (44%)	16 (100%)
**Total**						
CUS	1 (25%)	3 (75%)	4 (50%)	7 (54%)	6 (46%)	13 (76%)
QUS	0 (0%)	4 (100%)	4 (50%)	2 (50%)	2 (50%)	4 (24%)
Total	1 (13%)	7 (88%)	8 (100%)	9 (53%)	8 (47%)	17 (100%)
*p*-value	*p* = 1.00 *			*p* = 1.00 *		

CUS = Conventional up-dosing scheme/QUS = quick up-dosing scheme; * Fisher’s exact test comparing incidence of SRs in adolescents with adults.

**Table 5:** Change in health-related QoL assessment (SF-12) between first and last visits (no. of patients n = 86; adolescents: 8; adults: 78).

		Difference in PCS12 *				Difference in MCS12 *			
		Valid N	P25 **	Median	P75 **	Valid N	P25 **	Median	P75 **
**Adolescents**	CUS	4	−1.27	−0.41	1.06	4	−4.35	0.29	5.53
	QUS	4	−4.97	−2.65	−1.75	4	4.46	7.77	12.73
	Total	8	−2.65	−1.51	−0.41	8	−0.25	5.00	8.31
	*p*	0.043				0.149			
**Adults**	CUS	50	−2.13	0.04	1.70	50	−2.68	0.14	2.48
	QUS	28	−2.87	−0.65	2.51	28	−3.12	−0.79	2.55
	Total	78	−2.57	0.00	2.49	78	−3.01	0.00	2.48
	*p*	0.595				0.574			
**Total**	CUS	54	−1.96	0.00	1.70	54	−3.09	0.14	2.67
	QUS	32	−2.91	−0.94	2.10	32	−2.54	−0.05	4.27
	Total	86	−2.57	−0.11	1.71	86	−3.01	0.00	3.23
	*p*	0.280				0.754			

* Physical component score (PCS), mental component score (MCS)/** Percentile 25/Percentile 75/CUS = conventional up-dosing scheme/QUS = quick up-dosing scheme.

**Table 6:** QOL SF-12 domains in adolescents (n = 8): scores before and after 8 or 12 weeks of AIT treatment (end of observation period) show different outcomes depending on the up-dosing scheme.

	Physical Health (PCS)		Mental Health (MCS)	
Up-dosing scheme (no. of patients)	CUS n = 4	QUS n = 4	CUS n = 4	QUS n = 4
Baseline	42.9 ± 1.4	44.4 ± 1.9	42.8 ± 4.9	39.9 ± 1.7
End of observation period	42.8 ± 1.5	41.0 ± 2.3	43.4 ± 6.2	48.5 ± 3.7
*p* *	0.715	0.715	0.068	0.068
Up-dosing scheme (no. of patients)	CUS n = 50	QUS n = 28	CUS n = 50	QUS n = 28
Baseline	42.0 ± 4.7	41.8 ± 5.8	43.1 ± 5.5	44.9 ± 5.5
End of observation period	41.7 ± 4.8	42.1 ± 4.2	43.2 ± 5.5	45.3 ± 3.5
*p* *	0.888	0.680	0.683	0.792

CUS = Conventional up-dosing scheme/QUS = quick up-dosing scheme; * Wilcoxon signed rank test.

## Data Availability

Upon request, data will be available from the corresponding author.

## Supplementary Materials

The following supporting information can be downloaded at https://www.mdpi.com/article/10.3390/jcm14238456/s1: Table S1. General AE symptom distribution (n [%]) in the different study subgroups (adolescents and adults, plus subgroups for up-dosing schedules (conventional (CUS) and quick (QUS)). Table S2. Baseline demographic characteristics. Supplementary S1: Narrative SAR.

## Abbreviations

The following abbreviations are used in this manuscript:

ADR	Adverse Drug Reaction
AE	Adverse Event
AIT	Allergen ImmunoTherapy
AMG	German Medicines Act (Arzneimittelgesetz)
AR	Allergic Rhinitis
ARC	Allergic Rhinoconjunctivitis
CI	Confidence Interval
CRO	Clinical Research Organisation
CUS	Conventional Up-dosing Scheme
DBPC	Double-Blind Placebo-Controlled
EAACI	European Association of Allergy and Clinical Immunology
eCRF	Electronic Case Report Form
EDC System	Electronic Data Capture System
eDiary	Electronic patient Diary
ENCePP	European Network of Centres for Pharmacoepidemiology and Pharmacovigilance
IgE	Immunoglobulin E
ILIT	Intralymphatic ImmunoTherapy
LR	Local Reaction
MCS	Mental Component Score
NIS	Non-Interventional Study
OR	Odd’s Ratio
*p*	(*p*-value) value for significance
PASS	Post-Authorisation Safety Study
PCS	Physical Component Score
P25	Percentile 25
P75	Percentile 75
QoL	Quality of Life
QUS	Quick Up-dosing Scheme
SAE	Serious Adverse Event
SAR	Serious Adverse Reaction
SCIT	Subcutaneous ImmunoTherapy
SF-12	(Health survey) Short Form 12 questions
SLIT	SubLingual ImmunoTherapy
SmPC	Summary of Product Characteristics
SR	Systemic Reaction
V	Visit
WAO	World Allergy Organisation
