# Supplementary material for: Allergen Immunotherapy with Depigmented–Polymerised Cat Allergoid Is Safe and Well-Tolerated in Patients with Allergic Rhinitis/Rhinoconjunctivitis
Source: J Clin Med. 2025 Nov 28;14(23):8456. doi: 10.3390/jcm14238456 (PMC12692814; doi:10.3390/jcm14238456)
Supplement: Supplementary file 1 [file jcm-14-08456-s001.zip › jcm-3959286-supplementary.pdf]

Table S1. General AE symptoms distribution (n [%]) in the different study sub-groups. (adolescents, adults plus subgroups for up-dosing schedules (conventional (CUS) and quick (QUS)).

AE-Symptoms (n [%])	Adolescents			Adults			Total		
	**CUS**	**QUS**	**Total**	**CUS**	**QUS**	**Total**	**CUS**	**QUS**	**Total**
Cardiac disorders	0 (0%)	0 (0%)	0 (0%)	0 (0%)	1 (0.8%)	1 (0.3%)	0 (0%)	1 (0.7%)	1 (0.2%)
Ear and labyrinth disorders	0 (0%)	0 (0%)	0 (0%)	1 (0.4%)	0 (0%)	1 (0.3%)	1 (0.3%)	0 (0%)	1 (0.2%)
Eye disorders	1 (2.6%)	0 (0%)	1 (1.9%)	12 (4.3%)	3 (2.5%)	15 (3.8%)	13 (4.1%)	3 (2.2%)	16 (3.6%)
Gastrointestinal disorders	1 (2.6%)	2 (14.3%)	3 (5.8%)	2 (0.7%)	2 (1.6%)	4 (1.0%)	3 (1.0%)	4 (2.9%)	7 (1.6%)
General disorders and administration site conditions	21 (55.3%)	10 (71.4%)	31 (59.6%)	206 (74.6%)	71 (58.2%)	277 (69.6%)	227 (72.3%)	81 (59.6%)	308 (68.4%)
Infections and infestations	1 (2.6%)	0 (0.0%)	1 (1.9%)	5 (1.8%)	1 (0.8%)	6 (1.5%)	6 (1.9%)	1 (0.7%)	7 (1.6%)
Abnormal breathing	1 (2.6%)	0 (0.0%)	1 (1.9%)	0 (0.0%)	3 (2.5%)	3 (0.8%)	1 (0.3%)	3 (2.2%)	4 (0.9%)
Metabolism and nutrition disorders	0 (0.0%)	1 (7.1%)	1 (1.9%)	1 (0.4%)	0 (0.0%)	1 (0.3%)	1 (0.3%)	1 (0.7%)	2 (0.4%)
Nervous system disorders	3 (7.9%)	0 (0.0%)	3 (5.8%)	9 (3.3%)	3 (2.5%)	12 (3.0%)	12 (3.8%)	3 (2.2%)	15 (3.3%)
Psychiatric disorders	0 (0.0%)	1 (7.1%)	1 (1.9%)	0 (0.0%)	0 (0.0%)	0 (0.0%)	0 (0.0%)	1 (0.7%)	1 (0.2%)
Respiratory, thoracic and mediastinal disorders	10 (26.3%)	0 (0.0%)	10 (19.2%)	37 (13.4%)	38 (31.1%)	75 (18.8%)	47 (15.0%)	38 (27.9%)	85 (18.9%)
Skin and subcutaneous tissue disorders	0 (0.0%)	0 (0.0%)	0 (0.0%)	3 (1.1%)	0 (0.0%)	3 (0.8%)	3 (1.0%)	0 (0.0%)	3 (0.7%)
**Overall total (n)**	**38**	**14**	**52**	**276**	**122**	**398**	**314**	**136**	**450**

CUS = conventional up-dosing scheme / QUS = quick up-dosing scheme

Table S2. Baseline demographic characteristics (n=91).

Demographic characteristics			Adolescents (n=9)			Adults (n=82)			Total (n=91)		
			**CUS**	**QUS**	**Total**	**CUS**	**QUS**	**Total**	**CUS**	**QUS**	**Total**
**Gender**	**female**	n	3	4	7	31	21	52	34	25	59
		%	60.0%	100.0%	77.8%	60.8%	67.7%	63.4%	60.7%	71.4%	64.8%
	**male**	n	2	0	2	20	10	30	22	10	32
		%	40.0%	0.0%	22.2%	39.2%	32.3%	36.6%	39.3%	28.6%	35.2%
	**Total**	n	5	4	9	51	31	82	56	35	91
		%	100.0%	100.0%	100.0%	100.0%	100.0%	100.0%	100.0%	100.0%	100.0%
**Age**	**Valid N**		5	4	9	51	31	82	56	35	91
	**Mean**		14.40	14.75	14.56	35.43	36.39	35.79	33.55	33.91	33,69
	**Standard Deviation**		1.14	0.96	1.01	11.80	11.47	11.61	12.78	12.84	12.73

CUS = conventional up-dosing scheme / QUS = quick up-dosing scheme

Supplementary S1: Narrative SAR.

A systemic reaction in a 47-year-old female patient was categorised as grade 2 by the investigator and indicated as serious (SAR). The reaction occurred during the administration of the first injection with a dose of 0.1 mL and led to severe asthma, coughing and tingling of the palate after 15 minutes. The patient was administered with a salbutamol metered dose inhaler and given a 10 mg cetirizine tablet orally. This resulted in complete recovery of the patient's health state within 30 minutes. The patient was discharged as symptom-free, and AIT was continued according to the observation plan in the CUS group.
